# Identification of Sulfonamide-Vinyl Sulfone/Chalcone and Berberine-Cinnamic Acid Hybrids as Potent DENV and ZIKV NS2B/NS3 Allosteric Inhibitors

**DOI:** 10.3390/ijms262311762

**Published:** 2025-12-04

**Authors:** Panupong Mahalapbutr, Kowit Hengphasatporn, Wachirapol Manimont, Ladawan Vajarintarangoon, Yasuteru Shigeta, Nayana Bhat, Thitinan Aiebchun, Bodee Nutho, Supot Hannongbua, Thanyada Rungrotmongkol

**Affiliations:** 1 Department of Biochemistry, and Center for Translational Medicine, Faculty of Medicine, Khon Kaen University, Khon Kaen 40002, Thailand; 2 Center for Computational Sciences, University of Tsukuba, 1-1-1 Tennodai, Tsukuba, Ibaraki 305-8577, Japan; 3 Center of Excellence in Computational Chemistry (CECC), Department of Chemistry, Faculty of Science, Chulalongkorn University, Bangkok 10330, Thailand; 4 Center of Excellence in Structural and Computational Biology, Department of Biochemistry, Faculty of Science, Chulalongkorn University, Bangkok 10330, Thailand; 5 Department of Pharmacology, Faculty of Science, Mahidol University, Bangkok 10400, Thailand; 6 Program in Bioinformatics and Computational Biology, Graduate School, Chulalongkorn University, Bangkok 10330, Thailand

**Keywords:** molecular docking, molecular dynamics simulation, NS2B/NS3 serine protease, dengue virus, Zika virus

## Abstract

Dengue virus (DENV) and Zika virus (ZIKV) are flaviviruses transmitted by *Aedes* spp. mosquitoes, causing a spectrum of symptoms ranging from mild fevers and joint pain to severe damage to vital organs, including the kidneys, brain, and liver. Unfortunately, there are currently no specific treatments for these viruses. The NS2B/NS3 serine protease has been recognized as a crucial therapeutic target due to its pivotal role in viral replication. Herein, several molecular modeling techniques were employed to search for novel allosteric inhibitors against DENV and ZIKV NS2B/NS3 proteases from a set of 545 in-house compounds. Virtual screening based on molecular docking and MM/GBSA-based free energy calculations indicated that, among 545 derivatives, four compounds demonstrated high binding affinity against both targets, including two sulfonamide-vinyl sulfone hybrids (cpd48_e and cpd50_e), one sulfonamide-chalcone analog (cpd48), and one berberine-cinnamic acid derivative (DN071_f). Their molecular complexation was driven mainly by van der Waals forces rather than electrostatic attraction. Several residues at the enzyme allosteric site, particularly K74, L149, and N152 (DENV) and L76, I123, N152, and V155 (ZIKV), were identified as binding hotspots for the screened compounds. Drug-likeness predictions based on Lipinski’s rule of five further supported their potential as drug candidates. Overall, these findings provide valuable insights for the future design and development of novel antiviral drugs targeting the DENV and ZIKV NS2B/NS3 proteases.

## 1. Introduction

Vector-borne diseases pose a substantial public health burden, causing over 700,000 deaths each year and representing more than 17% of all infectious diseases [1,2]. *Aedes albopictus* and *Aedes aegypti* mosquitoes predominantly facilitate the transmission of dengue virus (DENV) and Zika virus (ZIKV) [3]. The global expansion of trade and travel, coupled with climate change, has led to the widespread dissemination of these mosquitoes, creating a conducive environment for the transmission of DENV and ZIKV, and the emergence of new epidemics [1]. Consequently, DENV and ZIKV infections have become a substantial global health concern, as epidemic outbreaks can have significant health consequences and incur substantial costs [1,4]. Both DENV and ZIKV infections exhibit similar symptoms, such as fever, headache, skin rashes, joint pains, and malaise [5]. However, the key distinction lies in the duration of the infections; ZIKV infection typically lasts for a few days or weeks before subsiding, while DENV infections may persist for several weeks and, if untreated, can result in severe complications, such as excessive bleeding and fatality [4]. Currently, there are no specific or effective antiviral drugs approved for the treatment of DENV and ZIKV infections [6]. Therefore, there is an urgent need to develop novel anti-flaviviral drugs that can effectively combat these two viruses to decrease the severity and fatality associated with these diseases.

**Figure 1:**
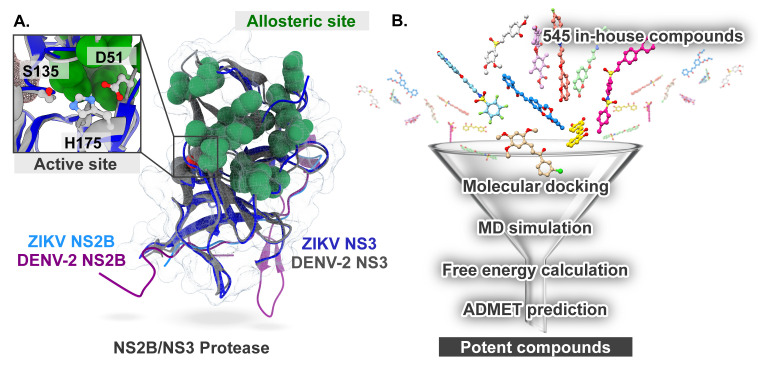
(**A**) Superimposed structures of DENV-2 and ZIKV NS2B/NS3 serine proteases showing the active site and allosteric region. (**B**) Computational workflow of this study.

A single-stranded positive-sense RNA genome of DENV and ZIKV encodes a single polyprotein that undergoes cleavage to form three structural proteins (capsid, precursor membrane/membrane, and envelope) and seven non-structural (NS) proteins (NS1, NS2A, NS2B, NS3, NS4A, NS4B, and NS5) by host and viral proteases [7]. Given that the NS2B/NS3 serine protease (Figure 1A) is mainly responsible for cleaving the viral polyprotein precursor to produce mature structural and NS proteins, it has become one of the attractive drug targets for the design and development of novel anti-flaviviral drugs [8,9].

However, a significant challenge in the initial stages of drug development against this target is the flat nature of the NS2B/NS3 active site, making the design of inhibitors through structure-based methods quite challenging [10]. Moreover, the highly negatively charged active site of NS2B/NS3 shows a preference for positively charged amino acids in peptide and peptidomimetic substrates. As a result, peptidomimetic inhibitors incorporating basic moieties exhibit poor cellular activity and pharmacokinetic properties, restricting their potential for advancement in preclinical development [11]. To overcome these difficulties, an alternative strategy is to identify and develop non-competitive inhibitors, specifically targeting the putative allosteric site located on the opposite side of the catalytic triad (His51–Asp75–Ser135) [10,12,13]. The NS2B/NS3 exists in two conformations: the inactive “super-open” conformation and the active “closed” conformation. During the transition from the closed to the super-open conformation, a transient allosteric pocket is exposed at the interface between NS2B and NS3 [14]. The binding of allosteric inhibitors potentially locks the protease in its inactive open conformation by preventing the conformational changes in the NS2B cofactor required to activate the NS3 protease [15].

In the present study, we aimed to investigate the binding mode and susceptibility of 545 in-house natural and semi-synthetic compounds containing chalcone, vinyl sulfone, sulfonamide, berberine, sulfonylated indenoquinoline, flavonoid, quinolinone, coumarin, evernic acid, cinnamic acid, and nitroolefin moieties against the DENV and ZIKV proteases using several molecular modeling techniques (Figure 1B). These compounds were previously designed and tested in our group against protein targets in cancer and type 2 diabetes [16,17,18,19,20]; however, their activity against DENV and ZIKV NS2B/NS3 has not been reported. Notably, previous studies have shown that chemical scaffolds such as chalcone [21], sulfonamide [22], berberine [23], flavonoid [24], coumarin [25], and cinnamic acid [26] can interact with viral NS2B-NS3 protease. Given their chemical backbones similar to the previously reported NS2B-NS3 protease inhibitors, we hypothesized that this library could be repurposed and systematically screened against DENV and ZIKV NS2B/NS3 protease.

Initially, the 545 in-house compounds underwent molecular docking toward both DENV and ZIKV proteases, alongside comparison with the known allosteric inhibitor, SYC-1307. The top-ranked compounds were subsequently subjected to molecular dynamics (MD) simulation and binding free energy calculation based on the molecular mechanics/generalized Born surface area (MM/GBSA) method to assess their structural stability and susceptibility against DENV and ZIKV proteases. Finally, the drug-likeness and pharmacokinetics of the screened compounds were evaluated. We hope that the potent allosteric inhibitors identified from this work will contribute to the development of effective antiviral drugs for the treatment of DENV and ZIKV infections.

## 2. Results and Discussion

### 2.1. Molecular Docking

**Figure 2:**
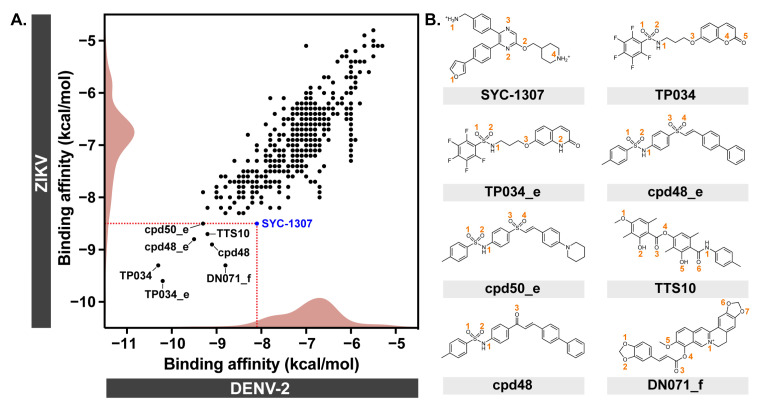
(**A**) Binding affinity of 545 in-house compounds and SYC-1307 inhibitor against DENV-2 and ZIKV NS2B/NS3 proteases. (**B**) Chemical structure of the top-ranked compounds, SYC-1307, TP034, TP034_e, cpd48_e, cpd50_e, TTS10, cpd48, and DN071_f.

Molecular docking results (Figure 2A) revealed that the binding affinity of the known inhibitor SYC-1307 against DENV-2 NS2B/NS3 (−8.1 kcal/mol) was similar to that against ZIKV NS2B/NS3 (−8.5 kcal/mol), which is in good agreement with the previous report showing that SYC-1307 can allosterically inhibit both ZIKV protease (IC_50_ = 0.20 ± 0.01 μM) and DENV-2 protease (IC_50_ = 0.59 ± 0.02 μM) [27]. Out of the 545 in-house derivatives, there were seven compounds that could bind to both viral proteases with docking energies in the range of −8.8 to −10.3 kcal/mol (DENV-2) and −8.5 to −9.6 kcal/mol (ZIKV), including TP034, TP034_e, cpd48_e, cpd50_e, TTS10, cpd48, and DN071_f (Table 1 and Figure 2B). Therefore, these seven derivatives were selected for further analyses in comparison to the SYC-1307 inhibitor.

### 2.2. System Stability and Water Accessibility

**Figure 3:**
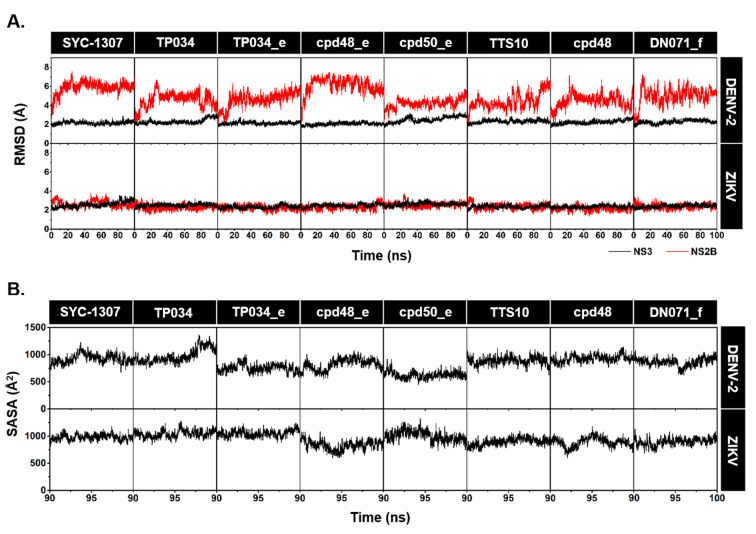
(**A**) Time evolution of RMSD of NS3 protease (black) and NS2B cofactor (red) of DENV-2 (top) and ZIKV (bottom) in complexes with SYC-1307, TP034, TP034_e, cpd48_e, cpd50_e, TTS10, cpd48, and DN071_f. (**B**) SASA within a 5.0 Å sphere of each ligand in complexes with DENV-2 (top) and ZIKV (bottom) NS2B/NS3 proteases.

The stability of NS2B/NS3–ligand complexes along the simulation times was investigated using the root-mean-square deviation (RMSD) calculation, and the obtained results are shown in Figure 3A. It should be noted that the N-terminal region of DENV-2 NS2B is longer than that of ZIKV NS2B, leading to the increased RMSD fluctuations (up to ~6–7 Å) as compared to ZIKV (~2–3 Å). However, the RMSD values for the NS3 protease of both DENV-2 and ZIKV, when bound to the eight studied ligands, exhibited a stable trend with minimal variations (~2–3 Å) over the entire simulations.

Given that the allosteric pocket of NS3 protease is a solvent-exposed area, the solvent-accessible surface area (SASA) within 5.0 Å sphere of each compound was calculated over the last 10 ns to reinforce the stability of the ligand binding. As depicted in Figure 3B, the SASA values consistently fluctuated within the range of 750–1000 Å for all studied systems, suggesting a high level of stability in the protein-ligand complexes. In this work, the MD trajectories from the last 10 ns (90–100 ns) of the simulations were selected for further analyses in terms of: (i) molecular mechanics energy (Δ*E*_MM_), (ii) binding free energy (Δ*G*_bind_), (iii) hydrogen bond (H-bond) formation, and (iv) per-residue decomposition free energy (Δ*G*_bind, residue_).

### 2.3. Binding Susceptibility

**Figure 4:**
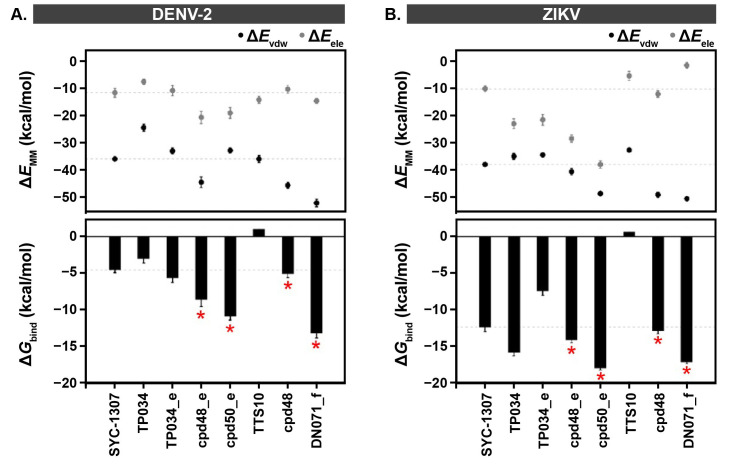
Δ*E*_MM_ (Top) and MM/GBSA-based Δ*G*_bind_ (Bottom) of SYC-1307, TP034, TP034_e, cpd48_e, cpd50_e, TTS10, cpd48, and DN071_f in complexes with (**A**) DENV-2 and (**B**) ZIKV proteases. Data are expressed as mean ± SEM (20 snapshots). The red asterisk (*) indicates compounds that exhibited high binding affinity against both targets.

**Figure 5:**
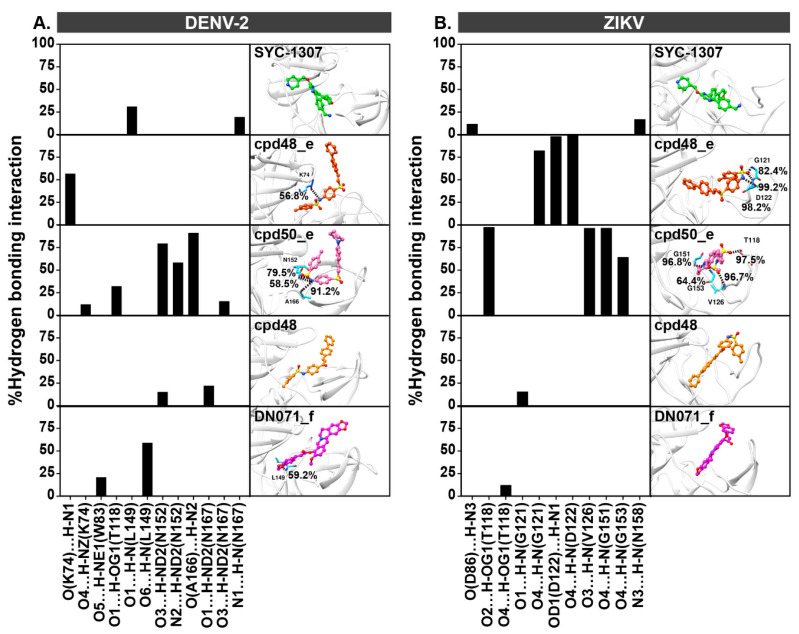
Percentage of H-bond occupation of (**A**) DENV-2 and (**B**) ZIKV proteases contributing to the binding of SYC-1307, cpd48_e, cpd50_e, cpd48, and DN071_f, where the ligand orientation in the enzyme allosteric site is illustrated in the right panel.

The binding affinity of the screened compounds against the DENV-2 and ZIKV NS2B/NS3 proteases was predicted using Δ*G*_bind_ calculations based on the MM/GBSA method. As depicted in Figure 4A,B, the Δ*E*_MM_ calculation in the gas phase demonstrated that van der Waals was the main force inducing protein-ligand complexation (Δ*E*_vdW_ of ~−25 to −55 kcal/mol) rather than electrostatic interaction (Δ*E*_ele_ of ~−2.5 to −40 kcal/mol). This finding is in accordance with previous reports indicating that the van der Waals interaction mainly contributed to the complexation between (i) the allosteric hits 1–10 and DENV-2 NS2B/NS3 protease [15], (ii) HIV/HCV inhibitors and DENV NS2B/NS3 [28], and (iii) compound 23/Cn-716 and ZIKV NS2B/NS3 protease [29]. The electrostatic contributions of cpd48_e and cpd50_e in complexes with both targets were higher than those of the other compounds, which agrees well with the H-bond analysis (Figure 5), as discussed later.

The MM/GBSA-based Δ*G*_bind_ results suggested that, among seven screened derivatives, there were five compounds that exhibited higher binding affinity against DENV-2 protease than the SYC-1307 inhibitor, including TP034_e, cpd48_e, cpd50_e, cpd48, and DN071_f (Figure 4A). Similarly, in the case of ZIKV protease, TP034, cpd48_e, cpd50_e, cpd48, and DN071_f showed higher susceptibility than the SYC-1307 inhibitor (Figure 4B). However, there were only four compounds, denoted by the red asterisk (*) that exhibited high binding affinity against both targets, including two sulfonamide-vinyl sulfone hybrids (cpd48_e and cpd50_e), one sulfonamide-chalcone analog (cpd48), and one berberine-cinnamic acid derivative (DN071_f). Of these, cpd50_e and DN071_f demonstrated the highest binding efficiency, suggesting their potential as promising allosteric inhibitors of DENV-2 and ZIKV NS2B/NS3 proteases. The replacement of the chalcone carbonyl group in cpd48 with a sulfonyl group in cpd48_e (vinyl sulfone) markedly increased its susceptibility to both proteins. This is consistent with a previous report showing that the vinyl sulfone moiety exhibits greater potency than its chalcone analog in activating the NRF2 signaling pathway and upregulating HO-1 gene expression [30]. The potential of sulfonamide, chalcone, berberine, and cinnamic acid derivatives as inhibitors of flavivirus NS2B/NS3 proteases was also evidenced by previous studies [23,26,31,32,33,34]. Altogether, the four screened sulfonamide-vinyl sulfone/chalcone and berberine-cinnamic acid hybrids (cpd48_e, cpd50_e, cpd48, and DN071_f) were selected for further structural and energetic analyses.

### 2.4. Hydrogen Bonding

The formation of H-bond is a crucial factor influencing the binding strength of protein-ligand complexes. In this work, H-bond interactions with values of >80%, 50–79%, and <50% were defined as strong, moderate, and weak H-bond interactions, respectively. In the case of DENV-2 NS2B/NS3 systems (Figure 5A), there were (i) one strong H-bond formation detected between O(A166) and H-N2 of cpd50_e (91.18%), (ii) four moderate H-bonds formed by O(K74)…H-N1 of cpd48_e (56.77%), N2 of cpd50_e…H-ND2(N152) (58.45%), O6 of DN071_f…H-N(L149) (59.14%), and O3 of cpd50_e…H-ND2(N152) (79.50%), and (iii) eight weak H-bonds generated by O4 of cpd50_e…H-NZ(K74) (13.18%), O3 of cpd50_e…H-ND2(N167) (15.64%), O3 of cpd48…H-ND2(N152) (17.73%), N1 of SYC-1307…H-N(N167) (19.50%), O5 of DN071_f…H-NE1(W83) (20.95%), O1 of cpd48…H-ND2(N167) (22.27%), O1 of SYC-1307…H-N(L149) (31.05%), and O1 of cpd50_e…H-OG1(T118) (32.23%)**.** The residues mentioned above also participated in the formation of H-bonds with the DENV-2 NS3 protease in various reported allosteric inhibitors targeting DENV-2 NS2B/NS3, including pyridine and pyrazine derivatives [15], adenosylhydrazine derivatives [35], ZINC000001680989 and ZINC000001679427 [36], as well as quercetin, rutin, and kaempferol 3-*O*-rutinoside [37].

According to ZIKV NS2B/NS3 models (Figure 5B), there were (i) six strong H-bonds formed by O4 of cpd48_e…H-N(G121) (82.40%), O3 of cpd50_e…H-N(V126) (96.67%), O4 of cpd50_e…H-N(G151) (96.75%), O2 of cpd50_e…H-OG1 (T118) (97.75%), OD1(D122)…H-N1 of cpd48_e (98.2%), and O4 of cpd48_e…H-N(D122) (99.20%), (ii) one medium H-bonds formed by O4 of cpd50_e…H-N(G153) (64.42%), and (iii) four weak H-bonds generated by O(D86)…H-N3 of SYC-1307 (11.8%), O4 of DN071_f…H-OG1(T118) (12.40%), O1 of cpd48…H-N(G121) (15.70%), and N3 of SYC-1307…H-N(N158) (17.00%). Similarly to our finding, the crucial involvement of G151 and G153 residues in H-bond formation was previously identified in compound 1 [38], hydroxychloroquine [39], tetrapeptide (d-RKOR), and 4-aminobenzamidine [40] in complexes with ZIKV NS2B/NS3.

Altogether, cpd50_e and cpd48_e exhibited a greater number of H-bond interactions with DENV-2 and ZIKV NS2B/NS3, which is consistent with their higher electrostatic contributions compared with the other ligands (Figure 4). Although DN071_f exhibited weak H-bond interactions with both proteases, its complexes showed the lowest van der Waals energies, contributing to the overall high binding affinity (Figure 4).

### 2.5. Key Binding Residues

**Figure 6:**
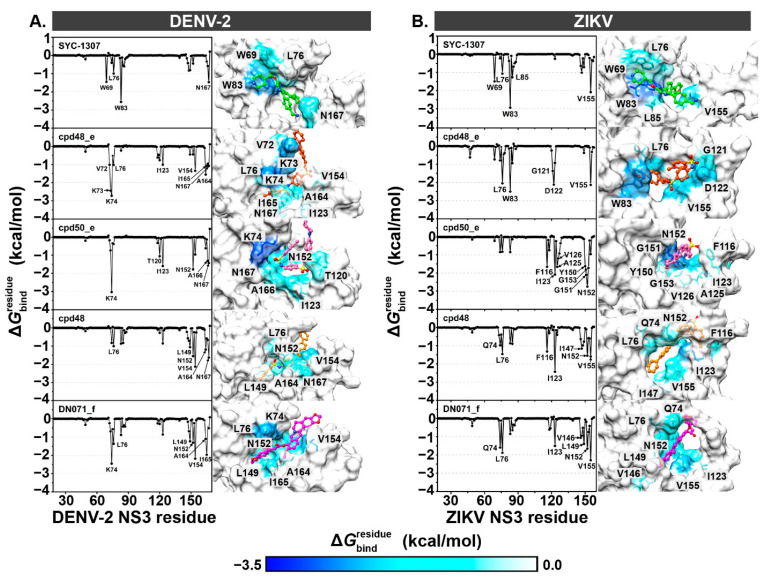
Δ*G*_bind, residue_ of (**A**) DENV-2 and (**B**) ZIKV NS2B/NS3 proteases in complexes with SYC-1307, cpd48_e, cpd50_e, cpd48, and DN071_f. Representative structures depicting the ligand orientation in the enzyme allosteric site, derived from the last MD snapshot, are presented to the right of each plot. The contributing residues involved in the ligand binding are colored according to their Δ*G*_bind, residue_ values, where the highest to lowest free energies are shaded from white to blue, respectively.

To identify the crucial NS3 residues associated with the ligand binding, the MM/GBSA-based Δ*G*_bind, residue_ calculation. It should be noted that (i) negative Δ*G*_bind, residue_ values represent ligand stabilization, whereas positive values indicate ligand destabilization, and (ii) residues exhibiting the Δ*G*_bind, residue_ of ≤−1.0 kcal/mol were considered in this analysis. The obtained results are illustrated in Figure 6. It has been reported that (i) DENV-2 NS3 residues, especially K74, L149, and N152 play a crucial role in interacting with allosteric inhibitors [35], (ii) targeting the N152 residue could stabilize the open conformation and impede the formation of a proteolytic β-hairpin structure [41], and (iii) interactions with K74 could alter enzyme’s catalytic activity due to ligand-induced conformational changes [42]. In strong accordance with these reports, the complexation of all the screened compounds revealed the presence of these three residues (Figure 6A). Additionally, W69, V72, K73, L76, W83, T120, I123, V154, A164, I165, A166, and N167 residues were also involved in the binding of our screened sulfonamide-vinyl sulfone/chalcone and berberine-cinnamic acid hybrids. Notably, the positively charged K74 residue showed the highest contribution in cpd48_e, cpd50_e, and DN071_f systems with the Δ*G*_bind, residue_ of ≤−2.4 kcal/mol, suggesting that these three compounds strongly interacted with the DENV-2 protease (Figure 4A).

In the case of ZIKV protease systems (Figure 6B), there were five (L76, W83, G121, D122, and V155), eight (F116, I123, A125, V126, Y150, G151, N152, and G153), seven (Q74, L76, F116, I123, I147, N152, and V155), and seven (Q74, L76, I123, V146, L149, N152, and V155) residues of ZIKV NS3 that were associated with the binding of cpd48_e, cpd50_e, cpd48, and DN071_f, respectively. Among these, the cpd50_e system exhibited the highest number of contributing residues, which was consistent with the Δ*G*_bind_ results (Figure 4B). These key residues have also been identified as binding hotspots in other reported ZIKV protease inhibitors [38,39,40,43,44].

### 2.6. Drug-Likeness and Pharmacokinetics

**Figure 7:**
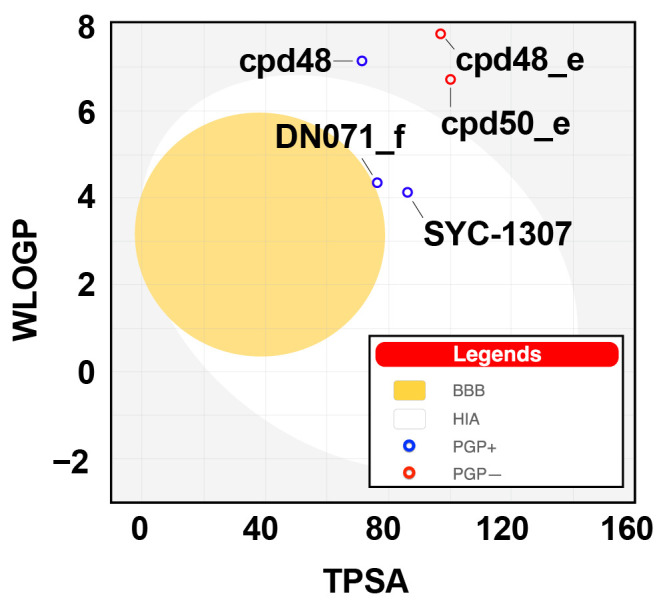
The Egan boiled-egg plot of SYC-1307, cpd48_e, cpd50_e, cpd48, and DN071_f. Gray region (lacking human intestinal absorption (HIA) and blood–brain barrier (BBB) access), white area (indicating HIA), and a yolk (indicating BBB access). WLOGP—lipophilicity, TPSA—topological polar surface area.

To further evaluate the drug-likeness of our screened sulfonamide-vinyl sulfone/chalcone and berberine-cinnamic acid hybrids (cpd48_e, cpd50_e, cpd48, and DN071_f) along with the SYC-1307 inhibitor, SwissADME web tool [45] was employed. As shown in Table 2, all predicted values for all investigated compounds fell within the range of Lipinski’s rule of five criteria: (i) molecular weight ≤ 500 Da, (ii) the number of H-bond donors ≤ 5 and the number of H-bond acceptors ≤ 10, (iii) the number of rotatable bonds ≤ 10, (iv) topological polar surface area ≤ 140 Å^2^, and (v) lipophilicity ≤ 5 [46]. The Egan boiled-egg model was further used to predict human intestinal absorption (HIA) and blood–brain barrier (BBB) permeation of the screened compounds. Results (Figure 7) showed that, among these four derivatives, only DN071_f could be passively absorbed by the gastrointestinal tract but was unable to permeate the BBB, which was similar to the SYC-1307 inhibitor. The SYC-1307, DN071_f, and cpd48 were predicted to be substrates of P-glycoprotein (P-gp), whereas cpd48_e and cpd50_e were not identified as P-gp substrates. Our screened compounds, especially DN071_f, exhibit drug-like characteristics, which could likely be further developed as antiviral drugs. However, SwissADME is designed mainly for drug-like organic molecules, making predictions less reliable for peptides or complex natural products. It cannot account for factors such as environmental sensitivity, and its accuracy decreases for molecules outside its applicability domain.

## 3. Computational Methods

### 3.1. Structural Preparation and Molecular Docking

The crystal structures of DENV-2 NS2B/NS3 protease (PDB ID: 2FOM [47]) and ZIKV NS2B/NS3 protease (PDB ID: 5GXJ) were obtained from the Protein Data Bank. The missing residues 77–84 in DENV-2 NS2B/NS3 protease were added using MODELLER software 10.6 implemented in UCSF Chimera 1.17.1. The chemical structures of 545 in-house compounds were obtained from our previous studies [16,17,18,19,20]. The ionizable amino acids of the proteins were predicted using PDB2PQR server at pH 7.4. Afterward, each compound was docked into the allosteric site [10,12,13] of DENV and ZIKV NS2B/NS3 proteases using AutoDock VinaXB (grid dimensions = 20Å × 20Å × 20Å; exhaustiveness = 16; number of runs = 10; empirical halogen bond scoring function [48]). Note that SYC-1307, an allosteric inhibitor of flavivirus NS2B/NS3 protease, was used as the reference ligand [27].

### 3.2. Molecular Dynamics (MD) Simulation

The chemical structures of the chosen ligands were optimized with the HF/6–31(d) level of theory using Gaussian09 program [49] as per previous reports [50,51,52]. The electrostatic potential (ESP) charges for the optimized ligands were computed using the same basis set. Afterward, the restrained ESP charges and missing parameters of each compound were achieved by the antechamber and parmchk modules of AMBER16. The ff14SB force field [53] was employed for the proteins, while the general AMBER force field [54] was applied for the ligands. The LEaP module of AMBER16 was used to add the missing hydrogens to the protein structures. The added hydrogen atoms were energy-minimized using the steepest descents (SD) and conjugated gradient (CG) methods. The complexes were solvated in the TIP3P water box [55] with a minimum distance of 13.0 Å from the protein surface. Counter ions were added to neutralize the simulated systems. Later, the water molecules were minimized using the SD and CG algorithms.

MD simulation was performed under the periodic boundary condition with a time step of 2 fs. The particle mesh Ewald (PME) summation method was used to treat the electrostatic interaction [56], while the cutoff of 12 Å was employed for non-bonded interactions. The SHAKE algorithm [57] was applied to constrain chemical bonds involving hydrogen atoms. The Berendsen barostat [58] was employed to maintain the system at standard pressure (1 atm), with a pressure-relaxation time of 1 ps. The target temperature (310 K) was controlled using the Langevin thermostat [59]. The simulations were performed for a duration of 100 ns.

### 3.3. Structural and Energetic Analyses

The CPPTRAJ module [60] of AMBER16 [61] was used to calculate the structural analyses, including RMSD, SASA, and H-bond. The H-bond formation was evaluated using two structural criteria: (i) distance between H-bond donor (HBD) and acceptor (HBA) ≤3.5 Å and (ii) the angle of HBD–H⋯HBA ≥ 120°. The MM/GBSA method [62] was utilized to calculate the Δ*G*_bind_ and Δ*G*_bind, residue_ of the complex structures on 20 frames extracted from the last 10 ns of the MD production phase.

### 3.4. Prediction of Drug-Likeness and Pharmacokinetics

Drug-likeness and pharmacokinetics of the screened compounds were predicted using the SwissADME webserver [45] as previously described [63,64]. The evaluation employed Lipinski parameters [46], a widely used set of criteria to estimate drug-likeness. The pharmacokinetics parameters predicted in this study include gastrointestinal absorption, P-glycoprotein substrate, and blood–brain barrier.

## 4. Conclusions

This work employed several molecular modeling techniques to search for novel allosteric inhibitors of DENV-2 and ZIKV NS2B/NS3 proteases from a set of 545 in-house compounds. Docking-based virtual screening demonstrated that, among 545 derivatives, only seven compounds (TP034, TP034_e, cpd48_e, cpd50_e, TTS10, cpd48, and DN071_f) that can bind to both viral proteases with the docking energies in the range of −8.5 to −10.3 kcal/mol, better than the known inhibitor, SYC-1307. The MM/GBSA-based Δ*G*_bind_ results suggested that there were four compounds that exhibited high binding affinity against both targets, including two sulfonamide-vinyl sulfone hybrids (cpd48_e and cpd50_e), one sulfonamide-chalcone analog (cpd48), and one berberine-cinnamic acid derivative (DN071_f). Among these, cpd50_e and DN071_f demonstrated the highest binding efficiency, suggesting their potential as promising allosteric inhibitors of DENV-2 and ZIKV NS2B/NS3 proteases. Their molecular complexation was driven mainly by van der Waals interactions rather than electrostatic attraction. The residues (i) W69, V72, K73, K74, L76, W83, T120, I123, L149, N152, V154, A164, I165, A166, and N167 of DENV-2 protease and (ii) Q74, L76, W83, F116, G121, D122, I123, A125, V126, V146, I147, L149, Y150, G151, N152, G153, and V155 of ZIKV protease were identified as binding hotspots for these four screened compounds. Predictions of drug-likeness based on Lipinski’s rule of five suggested that these compounds could possess characteristics associated with oral drug candidates. Taken together, the novel binding mechanisms identified for the four screened compounds, particularly DN071_f, could provide a foundation for the development of new allosteric inhibitors targeting DENV-2 and ZIKV NS2B/NS3 proteases.

## Figures and Tables

**Table 1:** Binding affinity of the top-ranked compounds against DENV-2 and ZIKV proteases.

		Binding Affinity (kcal/mol)	
Compound	Core Structure	DENV-2	ZIKV
SYC-1307	Reference	−8.1	−8.5
TP034	Sulfonamide, Coumarin	−10.3	−9.3
TP034_e	Sulfonamide, Quinolinone	−10.2	−9.6
cpd48_e	Sulfonamide, Vinyl sulfone	−9.5	−8.8
cpd50_e	Sulfonamide, Vinyl sulfone	−9.3	−8.5
TTS10	Evernic acid, 4-Methylaniline	−9.2	−8.7
cpd48	Sulfonamide, Chalcone	−9.1	−8.9
DN071_f	Berberine, 3,4-(Methylenedioxy)cinnamic acid	−8.8	−9.3

**Table 2:** Predicted values of drug-likeness parameters according to Lipinski’s rule of five criteria for SYC-1307, cpd48_e, cpd50_e, cpd48, and DN071_f.

Compound	Lipinski’s Rule of Five						Drug-Likeness
	MW (≤500 Da)	HBD (≤5)	HBA (≤10)	RB (≤10)	TPSA (≤140 Å^2^)	MLogP (≤5)	
SYC-1307	442.55	2	4	7	92.40	−5.53	Yes
cpd48_e	489.61	1	4	7	97.07	4.42	Yes
cpd50_e	496.64	1	4	7	100.31	3.48	Yes
cpd48	453.55	1	3	7	71.62	4.43	Yes
DN071_f	496.49	0	7	5	76.33	3.06	Yes

Note: MW—molecular weight; HBD—number of H-bond donors; HBA—number of H-bond acceptors; RB—number of rotatable bonds; TPSA—topological polar surface area; LogP—lipophilicity.

## Data Availability

The original contributions presented in this study are included in the article. Further inquiries can be directed to the corresponding authors.
